# Adherence to Mediterranean Diet and Cognitive Abilities in the Greek Cohort of Epirus Health Study

**DOI:** 10.3390/nu13103363

**Published:** 2021-09-25

**Authors:** Myrto Koutsonida, Afroditi Kanellopoulou, Georgios Markozannes, Styliani Gousia, Michail T. Doumas, Dimitrios E. Sigounas, Vasilios T. Tzovaras, Konstantinos Vakalis, Ioanna Tzoulaki, Evangelos Evangelou, Evangelos C. Rizos, Evangelia Ntzani, Eleni Aretouli, Konstantinos K. Tsilidis

**Affiliations:** 1 Department of Hygiene and Epidemiology, University of Ioannina School of Medicine, 45110 Ioannina, Greece; m.koutsonida@uoi.gr (M.K.); af.kanellopoulou@uoi.gr (A.K.); g.markozannes@uoi.gr (G.M.); itzoulak@cc.uoi.gr (I.T.); vangelis@uoi.gr (E.E.); entzani@uoi.gr (E.N.); 2 Laboratory of Cognitive Neuroscience, School of Psychology, Aristotle University of Thessaloniki, 54124 Thessaloniki, Greece; goustella@hotmail.com; 3 Ioannina Medical Care, 45333 Ioannina, Greece; michalis_doumas@yahoo.gr (M.T.D.); dsigounas@gmail.com (D.E.S.); vtzovi@yahoo.gr (V.T.T.); kosvak@yahoo.gr (K.V.); 4 Department of Epidemiology and Biostatistics, School of Public Health, Imperial College London, London W2 1PG, UK; 5 Department of Internal Medicine, University Hospital of Ioannina, 45500 Ioannina, Greece; vagrizos@gmail.com; 6 School of Medicine, European University of Cyprus, 2408 Nicosia, Cyprus; 7 Center for Evidence-Based Medicine, Department of Health Services, Policy and Practice, School of Public Health, Brown University, Providence, RI 02912, USA; 8 Institute of Biosciences, University Research Center of Ioannina, University of Ioannina, 45110 Ioannina, Greece; 9 School of the Social Sciences, University of Ioannina, 45500 Ioannina, Greece

**Keywords:** Mediterranean diet, cognition, middle-aged, cross-sectional, memory, executive functions, cognitive abilities, nutrition

## Abstract

The Mediterranean diet is commonly proposed as a major modifiable protective factor that may delay cognitive impairment in the elderly. The aim of the study was to investigate the cross-sectional association of adherence to the Mediterranean diet with cognitive abilities in a younger Greek population. A total of 1201 healthy adults aged 21–77 years (mean: 47.8) from the Epirus Health Study cohort were included in the analysis. Adherence to the Mediterranean diet was measured using the 14-point Mediterranean Diet Adherence Screener (MEDAS) and cognition was measured using the Trail Making Test, the Verbal Fluency test and the Logical Memory test. Statistical analysis was performed using multiple linear regression models adjusted for age, sex, education, body mass index, smoking status, alcohol consumption and physical activity. Overall, no association was found between the MEDAS score and cognitive tests, which could be explained by the young mean age and high level of education of the participants. Future studies should target young and middle-aged individuals to gain further understanding of the association between Mediterranean diet and cognition in this age group.

## 1. Introduction

The development of technology and the progress of medical science have led to an increase in life expectancy in recent years. This was accompanied with an increasing incidence of cognitive difficulties in the general population [1] that are, frequently, undetected and/or underestimated. As pharmacological approaches currently are not sufficient to treat cognitive problems, there is a strong interest in finding alternative preventive approaches, primarily aimed at lifestyle changes, as potentially modifiable risk factors.

An important factor that is under investigation is a healthy dietary pattern, commonly indicated by complying with guidelines of the Mediterranean diet. The Mediterranean diet is characterized by a high intake of extra virgin olive oil, vegetables, fruits, cereals, nuts and legumes, moderate intake of fish and other kinds of meat, dairy products and red wine, and a low intake of sweets and saturated fats [2].

The Mediterranean diet is thought to affect cognition through indirect cardiovascular, metabolic and inflammatory pathways [3,4]. The specific food elements that constitute the Mediterranean diet pattern are rich in nutrients such as monounsaturated and omega-3 polyunsaturated fatty acids, polyphenols, antioxidants, serum carotenoids, vitamins, fiber and magnesium. These nutrients have been found to improve lipid profiles and reduce blood pressure, insulin resistance, C-reactive protein and oxidative stress, which are markers that have been linked to cognitive decline [5,6,7,8]. As a result, the reduction of these markers leads to lower odds of cognitive impairment incidence.

Many studies have examined the association of the Mediterranean diet and cognitive function. Evidence from systematic reviews and meta-analyses tends to support the potential protective role of the Mediterranean diet on cognition [9,10,11,12]. However, some reviews have shown conflicting results [13,14], including a systematic review of five randomized control trials (RCTs) [14]. Moreover, while some studies may indicate positive associations between the Mediterranean diet and cognition, they fail to establish a relationship for most of the separate cognitive domains that were investigated [15,16,17,18,19].

It is possible that the reported results were affected by the country where each study was conducted, since most studies that report no association were conducted in non-Mediterranean populations [20,21,22]. The previous studies conducted in Greece that assessed Mediterranean dietary practices in relation to cognition have shown a positive association with cognitive performance and/or dementia [23,24,25,26,27,28], and there are only two studies that showed mixed results [29,30]. However, three of the six studies declaring positive associations have included participants from the same population of the Hellenic Longitudinal Investigation of Ageing and Diet (HELIAD) [23,27,28].

It is notable that the majority of the previously conducted studies as well as all of the aforementioned studies conducted in Greece included elderly participants over 60 years old. The limited studies that investigated the association of Mediterranean diet with cognition in young and middle-aged individuals reached inconsistent results. Two RCTs [15,19] comparing women that were randomly assigned for 10 days to either a Mediterranean style diet or no change diet found improvement in only one of the cognitive domains tested, memory and reaction time, accordingly. Additionally, there are two cross-sectional studies [21,22] that recruited participants aged 30–60 years and 20–70 years from non-Mediterranean countries, and only one of them found significant improvement in one of the cognitive domains tested (memory) [22], whereas the other observed no association [21]. Thus, the potential relation of the Mediterranean diet and cognition in middle-aged and younger adults remains unclear.

The goal of the current study was to investigate the cross-sectional association of the Mediterranean diet with cognition in a Greek sample of a mostly middle-aged population. We used data from the Epirus Health Study (EHS), a deeply phenotyped ongoing prospective cohort study in Greece comprising participants aged 21–77 years, with a relatively young mean age (47.8 years).

## 2. Materials and Methods

### 2.1. Study Population

The EHS (https://ehs.med.uoi.gr/, accessed on 4 August 2021) is an ongoing population-based prospective cohort study designed to investigate the etiology of multifactorial chronic diseases and to improve the overall health state of the Greek population. The study was approved by the Research Ethics Committee of the University of Ioannina and is conducted in accordance with the Declaration of Helsinki. All participants provided written informed consent prior to participation in the study.

**Figure 1:**
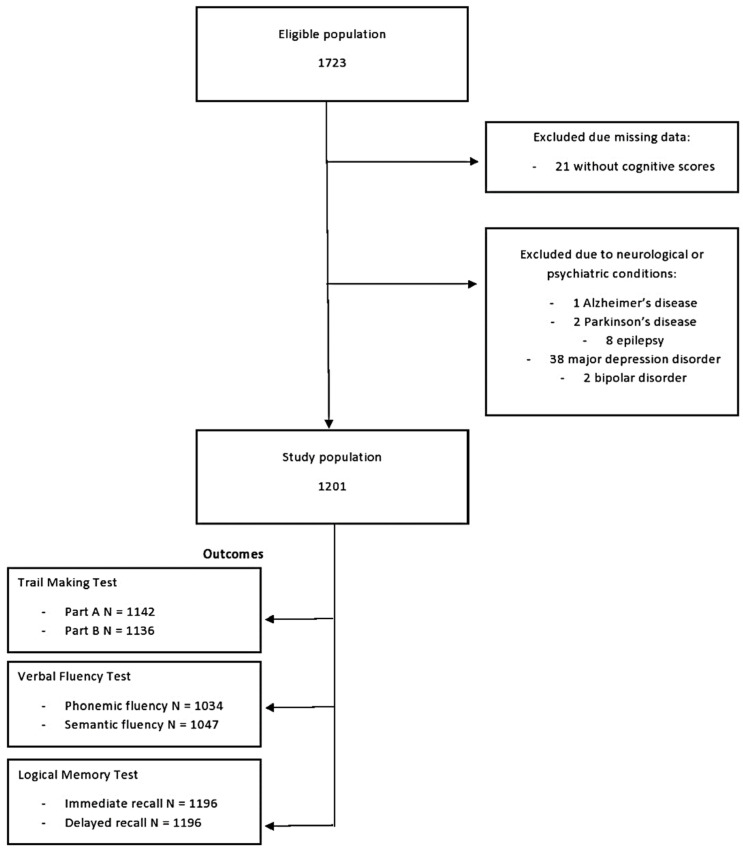
Flowchart for the identification of the study population. Each outcome was determined independent of the other outcomes.

The EHS cohort consists of permanent residents of the northwest region of Epirus in Greece. It was initiated in June 2019 and 1273 participants were recruited until April 2021. The vast majority of them were residents of the urban city of Ioannina and were fluent in the Greek language. For the purposes of the current study, we excluded 21 participants with missing data of cognitive scores and 51 participants who had self-reported serious neurological or psychiatric conditions at recruitment, namely 1 with Alzheimer’s disease, 2 with Parkinson’s disease, 8 with epilepsy, 38 with major depression disorder and 2 with bipolar disorder. The analyses were performed on the remaining 1201 participants, as outlined in Figure 1.

### 2.2. Data Collection

The EHS collects information on socio-demographic characteristics, lifestyle data, anthropometric, biochemical, clinical and cognitive measurements. A detailed description of the data collection procedure has been described previously [31]. Briefly, all participants were interviewed and examined by two trained medical professionals at recruitment. A standard questionnaire was used including questions for basic demographic characteristics (i.e., age, sex, place of birth, marital status, level of education, current employment status and income), personal and family medical history and lifestyle factors (i.e., physical activity, smoking habits, alcohol consumption, cellphone use). Weight, standing height and waist and hip circumference were measured using SECA equipment.

### 2.3. Adherence to the Mediterranean Diet

Adherence to the Mediterranean diet was estimated by calculating the 14-point Mediterranean Diet Adherence Screener (MEDAS), a brief screening questionnaire that has been found to be equivalent to a full-length validated food frequency questionnaire (FFQ) score [32]. MEDAS accounts for food habits and frequency of consumption for olive oil, vegetables, fruits, nuts, red and white meat, butter, sweets, soft drinks and wine. Each of the 14 questions is scored with 0 or 1, where one point was given for each target succeeded, generating a range of the total MEDAS score from 0 to 14, with a higher score indicating better Mediterranean diet adherence.

### 2.4. Cognitive Measurements

The standardized neuropsychological tests administered were the Greek versions of the Trail Making Test, the Verbal Fluency test and the Logical Memory test. These tests were chosen because there are used worldwide to assess cognition and they have sensitivity to detect cognitive decline even in preclinical stages [33,34]. Additionally, the available normative data for the Greek population are stratified by age and level of education, providing useful cut-off values suggestive of cognitive decline that take into consideration the impact of these variables.

The Trail Making Test (TMT) [35] is a test of attention and executive cognition, which consists of two parts. In the first part (Trail Making-Part A–TMT-A), participants were in-structed to connect a set of dots with numbers in an ascending order as quickly as possible while maintaining accuracy, and in the second part (Trail Making-Part B–TMT-B), to connect a set of dots with numbers and letters in an ascending pattern with the added task of switching between the numbers and letters while maintaining accuracy. Scoring was based on seconds needed to complete the test. Lower scores indicated better cognitive performances. An additional score, the difference between TMT-A and TMT-B, which represents the flexibility of the cognitive system independently from motor speed, was included in the analysis.

The Verbal Fluency test (VF) [36] is a measure of executive cognition that also relies on a language component. Participants were asked to generate as many words as possible from a given category in 60 s. This category could be semantic (semantic fluency subtest), including words belonging to the same semantic category (animals), or phonemic (phonemic fluency subtest), including words beginning with a specified letter (Greek letter “X”). The total score was the sum of the words produced in each subtest.

The Logical Memory test (LM) [37] is a verbal episodic memory test. Participants were read a story and asked to recite the story from memory immediately after its presentation (immediate recall condition). The procedure of immediate recall was repeated once, so that a learning curve was achieved. Approximately 20 min later, participants were asked again to recall the story (delayed recall condition). The number of correctly recalled information compiled the total score, ranging from 0–32 for the immediate recall condition and 0–16 for the delayed recall condition.

### 2.5. Statistical Analysis

Baseline characteristics of the study sample were summarized using means and standard deviations (SD) or percentages, as appropriate for continuous and categorical variables, respectively. Baseline characteristics were analyzed according to MEDAS and cognition tests using independent sample t-tests or x^2^ and Fisher’s exact tests.

Multiple linear regression analysis was used to investigate the cross-sectional association of adherence to the Mediterranean diet, as measured by the MEDAS score and the continuous scores derived from the three neuropsychological cognitive tests. Ordinal and logistic regression analyses were also performed to determine whether adherence to the Mediterranean diet was associated with odds of cognitive impairment using the transformed categorical scores of the aforementioned variables. Specifically, continuous scores of the neuropsychological tests were transformed to three categories (normal performance defined as scores at the mean value and above, borderline performance defined as scores up to 1.5 standard deviation below the mean value and abnormal performance defined as scores lower than 1.5 standard deviation below the mean) or binary scores (normal performance defined as performance better than 1.5 standard deviation below the mean value, and abnormal performance defined as scores lower than 1.5 standard deviation below the mean), based on existing normative cut-off scores [35,36,37] that are suggestive of cognitive decline after accounting for the age and education level of each participant. Continuous scores of MEDAS were also transformed based on the proposed cut-off score for lower level of adherence (0–7 points) and higher level of adherence (8–14 points) [32].

Furthermore, a composite z-score score was calculated and used as an indicator of overall cognition. Each score of every cognitive test was first converted into z-scores using mean and standard deviation values of published normative data [35,36,37]. Then, z-scores of both conditions of each neuropsychological test were averaged to create domain z-scores of executive cognition (using reverse scores of Trail Making Test-part A and B conditions), memory (using scores of Logical Memory test, immediate recall condition and delayed recall condition) and verbal fluency (using scores of Verbal Fluency test, semantic fluency condition and phonemic fluency condition). Finally, domain z-scores were averaged to produce the composite z-score. A higher z-score indicates better cognitive performance.

A sensitivity analysis was performed after excluding participants that did not have Greek as their first language (N = 104) to control for possible language difficulties that could affect the performance on cognitive tests, but the results were very similar to the main analysis and are not further shown herein.

All models were adjusted for age (continuous), sex and education (primary and secondary school, high school, higher education), and additionally adjusted for body mass index (BMI; continuous), smoking status (current, former or never smokers), alcohol consumption (never, less than once/month, 1–3 times/month, 1–2 times/week, almost every day) and recreational physical activity (measured in Metabolic Equivalents of Energy Expenditure (METs) per hour/week) (continuous).

Interaction analyses of MEDAS score with age groups (young: <40 years, middle-aged: 40–59.9 years, old: >60 years) and MEDAS score with sex were performed in the fully adjusted model to examine if the associations could be modified by these factors, as indicated by previous studies [29,38]. For the significant interaction results, stratified analyses by age groups were performed to identify the underlying associations.

All statistical analyses were performed using STATA (version 14; StataCorp, College Station, TX, USA). A *p*-value of less than 0.05 was considered as statistically significant for all analyses and a *p*-value of less than 0.10 was considered suggestive for the interaction analyses.

## 3. Results

Table 1 presents the sociodemographic characteristics of the study participants over-all and by the binary MEDAS score. Overall, the mean age was 47.82 (SD = 11) years, with a range of 21 to 77 years, and women (59.45%) and individuals of high education (67%) predominated in the sample. The mean MEDAS score was 7.25 (SD = 1.74) with a range of 2 to 13, suggesting a general moderate adherence to the Mediterranean diet. The mean BMI was 26.41 (SD = 4.68) kg/m^2^. Approximately one in three participants were current smokers, and 41.13% drank alcohol at least once per week.

Participants with higher adherence to the Mediterranean diet were older, more often women, had lower BMI, were less likely to be current smokers and were more physically active, in comparison with participants with lower adherence.

Supplementary Tables S1–S6 summarize the characteristics of included participants by the binary scores of each of the six cognitive tests. In all cognitive tests, MEDAS score tended to be lower in the group of participants with abnormal performance (with the only exception of the Verbal Fluency test, phonemic subtest) and BMI greater in the group of participants with abnormal performance (with the exception of both conditions of the Trail Making Test). Regarding demographic characteristics, participants with normal performance were more often women and more highly educated (except for the Trail Making Test-Part A and the Verbal Fluency test, phonemic subtest).

No significant associations were observed after evaluating the associations between continuous scores of MEDAS and cognitive tests (Table 2). Using ordinal or binary terms for the neuropsychological cognitive tests did not alter the results (Supplementary Tables S7 and S8), nor when binary categories of the MEDAS scores were used for any outcome term (Supplementary Tables S9 and S10). A significant association was found only between the bi-nary score of the Verbal Fluency test, semantic subtest, and continuous MEDAS score (*p* = 0.031), but it was attenuated in the fully adjusted model (*p* = 0.049, Supplementary Table S8).

**Figure 2:**
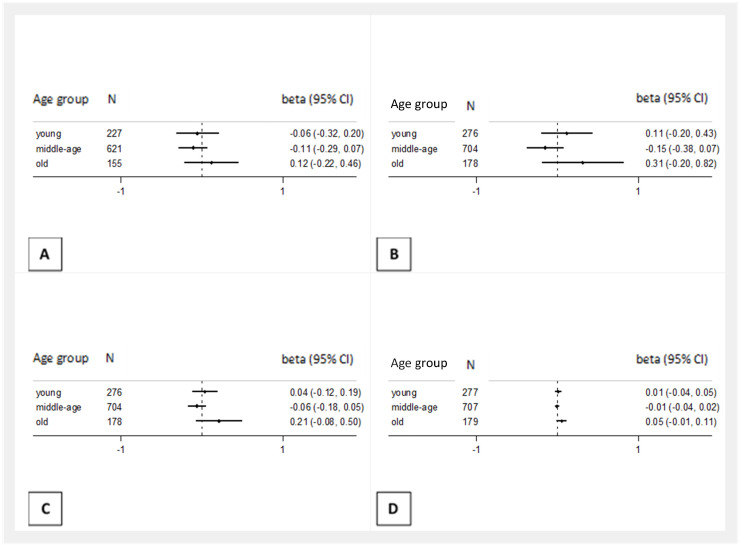
Results from multiple linear regressions for the association between adherence to the Mediterranean diet and the continuous scores of the four cognitive tests, stratified by age among Epirus Health Study participants. Models were adjusted for age (continuous), sex, education, BMI, smoking status, alcohol consumption and physical activity. (**a**) Verbal fluency, phonemic subtest, (**b**) Logical Memory, immediate recall, (**c**) Logical Memory, delayed recall, and (**d**) composite z-score. Young: <40 years, middle-aged: 40–59.9 years, old: >60 years.

When multiple linear regression analyses using the interaction terms by age and sex were performed, there was little evidence for interaction for most cognitive function scores (Table 3). Exceptions were the significant or suggestive interactions of MEDAS with age in relation to the Verbal Fluency, phonemic subtest (P-interaction 0.040), the Logical Memory, immediate recall (P-interaction 0.033), the Logical Memory, delayed recall (P-interaction 0.076) and the composite z-score (P-interaction 2.000^−14^). When the models were run by age groups, the associations of MEDAS with the cognitive tests were not statistically significant in any age group. There was a suggestion of a positive association in the old age group (>60 years), where the point estimates were positive but with wide confidence intervals (Pphonemic = 0.480, Pimmediate recall = 0.231, Pdelayed recall = 0.134, Pz-score = 0.114) (Figure 2).

## 4. Discussion

The results of the present cross-sectional analysis in the Greek Epirus Health Study cohort did not provide support for an association of adherence to the Mediterranean diet with cognitive abilities. These results are in line with some previous cross-sectional [20,21,22] and longitudinal studies [39,40,41,42,43,44,45] that reported a lack of association between the Mediterranean diet and cognition. It is noteworthy that almost half of these studies included middle-aged participants from non-Mediterranean countries [21,22,41,45]. Similarly, some RCTs reported findings compatible with our results, namely a lack of evidence of a beneficial effect of a Mediterranean diet intervention on cognitive function in all [46] or in most of the cognitive domains examined [15,19], whereas other RCTs found that participants allocated to Mediterranean diet intervention groups improved their cognitive abilities [47,48]. A systematic review of these RCTs concluded that there was large between-trial variation regarding the Mediterranean diet interventions and that the results had small effect sizes and were insignificant for most of the investigated cognitive domains [14]. However, these results are in contrast with the majority of past research that highlight adherence to the Mediterranean diet as either a significant positive correlate of cognition cross-sectionally [23,24,49,50] and longitudinally [51,52,53,54,55], or as a negative correlate of cognitive impairment cross-sectionally [56] and longitudinally [25,26,57,58,59].

A possible explanation for this discrepancy could be attributed to the fact that younger and highly educated people were included in the current study. It is hypothesized that the potential positive association of the Mediterranean diet with cognition is mediated by its protective role on cardiovascular, metabolic and inflammatory risk factors, such as blood pressure, total serum cholesterol and C-reactive protein, which in turn help maintain brain health and functioning [3,4]. As a result, longer periods of adherence are needed for the favorable association of the Mediterranean diet to be manifested in cognitive function by alleviating the aforementioned risk factors and preserving an optimum level of brain function. In line with this interpretation, some previous studies failed to establish an association between the Mediterranean diet and cognitive performances in participants younger than 65 years old, whereas they did observe an association for participants older than 65 years old [38,60]. In addition, the high level of education of our study participants is considered to delay the cognitive decline through cognitive reserve theory [61,62].

Another possible explanation for the discrepancy between previous studies and the current investigation could be the different methodology of cognitive assessment and the assessment of Mediterranean diet adherence. A large proportion of previous studies that reported associations between adherence to the Mediterranean diet and cognition assessed cognition with the Mini Mental State Examination (MMSE) [24,25,47,49,50,55,58,59]. However, MMSE is a screening test of global cognition and is not considered sensitive to assess cognitive abilities in specific cognitive domains [63]. On the contrary, in the present study, we used domain-specific cognitive tests that assessed executive cognition, memory and language. In fact, previous studies that administered domain-specific tests found positive associations of the Mediterranean diet when all cognitive domains were combined as a global score [23,26,48,51,53,54,56]. However, only one of those studies have found positive associations with all cognitive domains separately [51], and the rest report non-significant associations for certain cognitive domains [23,26,48,53], in line with the results of the present study [23,48].

Similarly, adherence to the Mediterranean diet in the present study was measured using the 14-point MEDAS score, whereas the 55-point Mediterranean Dietary Score (MedDietScore) [23,24,26,53,54,56] or the 9-point Mediterranean Diet Score (MDS) [25,49,52,57,59] were used in previous investigations. Each score may assess different aspects of adherence to the Mediterranean diet, further complicating the comparison across studies.

Nevertheless, some individual associations were found in the current study. Namely, the MEDAS score was associated with the binary score of the Verbal Fluency, semantic subtest. In addition, significant interactions of MEDAS score and age groups were observed for the Verbal Fluency, phonemic subtest, the Logical Memory, immediate and delayed recall, and the composite z-score, and there was a suggestion of a positive association in the old age group (>60 years), with point estimates being positive in this age group but with wide confidence intervals.

Our study has several strengths. To the best of our knowledge, this is one of a few studies that have examined the association of adherence to the Mediterranean diet with cognition in middle-aged individuals. Furthermore, standardized domain-specific neuropsychological tests with age-specific and education-specific normative data were used, increasing the accuracy of the measurements.

Several limitations should also be considered in the interpretation of our findings. The cross-sectional design of the study precludes any inference of causality. Additionally, we were unable to investigate the broader concept of healthy lifestyle patterns based on the combination of the Mediterranean diet with other lifestyle factors, including sleep quality and social interaction [27,28,64], an area that has gained increased interest in recent years. Finally, most of our participants were of younger age and of high education compared to previous studies, limiting the comparability and generalizability of the present results.

## 5. Conclusions

The results of the present study suggest that adherence to the Mediterranean diet is not associated with cognitive abilities in a Greek study of mostly middle-aged participants. Based on the existence of a plausible biological mechanism through which the Mediterranean diet could prevent or delay cognitive decline, further studies are needed to clarify the corresponding association in young and middle-aged populations.

## Figures and Tables

**Table 1:** Sociodemographic and lifestyle characteristics of Epirus Health Study participants by level of adherence to the Mediterranean diet.

Characteristics	All Participants (*n* = 1201)	Adherence to Mediterranean Diet ^†^		*p*-Value
		Low (*n* = 650)	High (*n* = 551)	
Age	47.82 ± 11.00	45.53 ± 10.33	50.53 ± 11.17	2.106^−15 a^
Female	714 (59.45)	364 (56.00)	350 (63.52)	0.008 ^b^
Education				0.688 ^b^
Primary and secondary school *	88 (7.33)	44 (6.77)	44 (8.00)	
High school **	308 (25.67)	170 (26.15)	138 (25.09)	
Higher education ***	804 (67.00)	436 (67.08)	368 (66.91)	
MEDAS score	7.25 ± 1.74	5.95 (1.10)	8.79 (0.92)	1.781^−282 a^
BMI	26.41 ± 4.68	26.74 ± 4.78	26.01 ± 4.54	0.008 ^a^
Smoking status				0.016 ^b^
Non-smokers	534 (44.46)	293 (45.08)	241 (43.74)	
Former smokers	286 (23.81)	135 (20.77)	151 (27.40)	
Current smokers	381 (31.72)	222 (34.15)	159 (28.86)	
Alcohol consumption				0.074 ^b^
Never	148 (12.32)	85 (13.08)	63 (11.43)	
Less than once/month	354 (29.48)	185 (28.46)	169 (30.67)	
1–3 times/month	205 (17.07)	121 (18.62)	84 (15.25)	
1–2 times/week	337 (28.06)	188 (28.92)	149 (27.04)	
Almost every day	157 (13.07)	71 (10.92)	86 (15.61)	
Physical activity (METs-hours/week)	15.58 ± 20.33	13.52 ± 18.18	18.02 ± 22.37	0.001 ^a^

Abbreviations: BMI: body mass index, METs: Metabolic Equivalents of Energy Expenditure. ^†^ Adherence to the Mediterranean diet was assessed using the Mediterranean Diet Adherence Screener (MEDAS). Low and high adherence were defined if MEDAS ranged between 0–7 and 8–14, respectively. * Elementary school or junior high school, up to 9 years of education. ** High school, up to 12 years of education. *** University degree/MSc/PhD/Postdoc, more than 13 years of education. ^a^ Comparisons using t-test. ^b^ Comparisons using x^2^ test. Mean ± standard deviation and frequency (percentage) are presented for continuous and categorical variables, respectively.

**Table 2:** Results of multiple linear regressions for the association between adherence to the Mediterranean diet (continuous score) and cognitive function (continuous score) among Epirus Health Study participants.

Cognitive Functions Scores	Adherence to Mediterranean Diet ^†^			
	Model 1 ^a^		Model 2 ^b^	
	Beta	95% CI	Beta	95% CI
Trail Making Test (Part A)	0.133	−0.254, 0.519	0.043	−0.357, 0.443
Trail Making Test (Part B)	−0.024	−0.591, 0.544	−0.103	−0.692, 0.486
Trail Making Test (Part B-A)	−0.242	−0.734, 0.245	−0.234	−0.745, 0.276
Verbal Fluency (semantic)	0.123	−0.081, 0.326	0.097	−0.116, 0.310
Verbal Fluency (phonemic)	−0.031	−0.160, 0.098	−0.060	−0.196, 0.074
Logical Memory (immediate recall)	0.023	−0.142, 0.189	−0.010	−0.183, 0.164
Logical Memory (delayed recall)	0.038	−0.049, 0.124	0.007	−0.082, 0.097
Composite z-score	0.010	−0.013, 0.033	0.006	−0.018, 0.029

Abbreviation: CI: Confidence interval. ^†^ Adherence to the Mediterranean diet was assessed using the Mediterranean Diet Adherence Screener (MEDAS). MEDAS score analyzed as continuous score (0–14). ^a^ Adjusted for age (continuous), sex, education. ^b^ Adjusted for age (continuous), sex, education, BMI, smoking status, alcohol consumption and physical activity.

**Table 3:** Results of multiple linear regressions for the association between adherence to the Mediterranean diet and cognitive function (continuous score) with the interaction terms by age and sex among Epirus Health Study participants.

Cognitive Functions Scores	Adherence to Mediterranean Diet					
	Interaction with Age			Interaction with Sex		
	Beta	95% CI	*p*-Value	Beta	95% CI	*p*-Value
Trail Making Test (Part A)	−0.052	−0.332, 0.227	0.714	0.249	−0.508, 1.006	0.519
Trail Making Test (Part B)	−0.212	−0.623, 0.199	0.312	0.285	−0.829, 1.399	0.616
Trail Making Test (Part B-A)	−0.221	−0.577, 0.135	0.224	−0.035	−1.000, 0.931	0.944
Verbal Fluency (semantic)	−0.000	−0.150, 0.150	0.996	0.044	−0.354, 0.443	0.828
Verbal Fluency (phonemic)	0.099	0.004, 0.194	0.040	−0.106	−0.359, 0.146	0.410
Logical Memory (immediate recall)	0.132	0.011, 0.252	0.033	0.214	−0.115, 0.544	0.201
Logical Memory (delayed recall)	0.057	−0.006, 0.119	0.076	0.071	−0.099, 0.241	0.414
Composite z-score	0.063	0.047, 0.078	2.000^−14^	.001	−0.043, 0.045	0.967

Abbreviation: CI: Confidence interval. Models adjusted for age (continuous), sex, education, BMI, smoking status, alcohol consumption, physical activity.

## Data Availability

Data are available upon reasonable request to the corresponding author.

## Supplementary Materials

The following are available online at www.mdpi.com/article/10.3390/nu13103363/s1, Table S1: Sociodemographic and lifestyle characteristics of Epirus Health Study participants by binary categories of Trail Making-Part A scores, Table S2: Sociodemographic and lifestyle characteristics of Epirus Health Study participants by binary categories of Trail Making-Part B scores, Table S3: Socio-demographic and lifestyle characteristics of Epirus Health Study participants by binary categories of Verbal Fluency, semantic subtest scores, Table S4: Sociodemographic and lifestyle characteristics of Epirus Health Study participants by binary categories of Verbal Fluency, phonemic subtest scores, Table S5: Sociodemographic and lifestyle characteristics of Epirus Health Study participants by binary categories of Logical Memory, immediate recall scores, Table S6: Sociodemographic and lifestyle characteristics of Epirus Health Study participants by binary categories of Logical Memory, delayed recall scores, Table S7: Results of ordinal logistic regression for the associations between adherence to the Mediterranean diet (continuous score) and cognitive function (ordinal score) among Epirus Health Study participants, Table S8: Results of logistic regression for the associations between adherence to the Mediterranean diet (continuous score) and cognitive function (binary score) among Epirus Health Study participants, Table S9: Results of multiple linear regression for the associations between adherence to the Mediterranean diet (binary score) and cognitive function (continuous score) among Epirus Health Study participants, Table S10: Results of logistic regression for the associations between adherence to the Mediterranean diet (binary score) and cognitive function (binary score) among Epirus Health Study participants.
