# Supplementary material for: Adherence to Mediterranean Diet and Cognitive Abilities in the Greek Cohort of Epirus Health Study
Source: Nutrients. 2021 Sep 25;13(10):3363. doi: 10.3390/nu13103363 (PMC8541267; doi:10.3390/nu13103363)
Supplement: Supplementary file 1 [file nutrients-13-03363-s001.zip › nutrients-1348781-supplementary Table S1.pdf]

Supplementary Table S1. Sociodemographic and lifestyle characteristics of Epirus Health Study participants by binary categories of Trail Making-Part A scores.

Variables	Trail Making-Part A binary score		p value
	**Normal performance (n= 1.111)**	**Abnormal performance (n=31)**	
Age	47.83 ± 10.66	43.00 ± 14.01	0.014 [a]
Female	655 (68.75)	21 (67.74)	0.326 [b]
Education			0.049 [c]
Primary and secondary school*	77 (6.93)	0 (0)	
High school**	293 (26.37)	4 (12.90)	
Higher education***	741 (66.70)	27 (87.10)	
MEDAS score	7.24 ± 1.75	7.16 ± 1.77	0.809 [a]
BMI	26.42 ± 4.70	25.22 ± 4.37	0.161 [a]
Smoking status			0.616 [c]
Non-smokers	493 (44.37)	16 (51.61)	
Former smokers	263 (23.67)	5 (16.13)	
Current smokers	355 (31.95)	10 (32.26)	
Alcohol consumption			0.337 [c]
Never	138 (12.42)	2 (6.45)	
Less than once/month	328 (29.52)	12 (38.71)	
1-3 times/month	182 (16.38)	6 (19.35)	
1-2 times/week	313 (28.17)	10 (32.26)	
Almost every day	150 (13.50)	1 (3.32)	
Physical activity (METs)	15.54 ± 20.69	15.37 ± 16.57	0.964 [a]

Abbreviations: BMI; Body mass index, METs; Metabolic Equivalents of Energy Expenditure
*Elementary school or junior high school, up to 9 years of education. **High school, up to 12 years of education. ***University degree/MSc/PhD/Postdoc, more than 13 years of education.
[a] Comparisons using t-test. [b] Comparisons using x^2 test. [c] Comparison's using Fisher's exact test.
Mean ± standard deviation and frequency (percentage) are presented for continuous and categorical variables, respectively.
