# Supplementary material for: Adherence to Mediterranean Diet and Cognitive Abilities in the Greek Cohort of Epirus Health Study
Source: Nutrients. 2021 Sep 25;13(10):3363. doi: 10.3390/nu13103363 (PMC8541267; doi:10.3390/nu13103363)
Supplement: Supplementary file 1 [file nutrients-13-03363-s001.zip › nutrients-1348781-supplementary Table S10.pdf]

Supplementary Table S10. Results of logistic regression for the associations between adherence to Mediterranean diet (binary score) and cognitive function (binary score) among Epirus Health Study participants.

**Cognitive scores†**	**Adherence to Mediterranean diet ‡**			
	**Model 1[a]**		**Model 2[b]**	
	**OR**	**95% CI**	**OR**	**95% CI**
Trail Making Test (Part A)	0.799	0.369, 1.730	0.842	0.380, 1.865
Trail Making Test (Part B)	0.333	0.069, 1.597	0.274	0.054, 1.397
Verbal Fluency (semantic)	0.590	0.333, 1.045	0.627	0.345, 1.139
Verbal Fluency (phonemic)	1.072	0.800, 1.436	1.136	0.839, 1.539
Logical Memory (immediate recall)	1.330	0.735, 2.408	1.473	0.796, 2.725
Logical Memory (delayed recall)	0.898	0.407, 1.981	0.966	0.422, 2.215

[a] Adjusted for age, gender, education level. [b] Adjusted for age, gender, education level, BMI, smoking status, alcohol consumption and physical activity.

† Cognitive scores analyzed as binary score (normal performance defined as performance better than 1.5 standard deviation below the mean value, and abnormal performance defined as scores lower than 1.5 standard deviation below the mean)

‡Adherence to Mediterranean diet was assessed using the Mediterranean Diet Adherence Screener (MEDAS). MEDAS score analyzed as binary categories of low and high adherence, ranged between 0-7 and 8-14, respectively.
