# Supplementary material for: Adherence to Mediterranean Diet and Cognitive Abilities in the Greek Cohort of Epirus Health Study
Source: Nutrients. 2021 Sep 25;13(10):3363. doi: 10.3390/nu13103363 (PMC8541267; doi:10.3390/nu13103363)
Supplement: Supplementary file 1 [file nutrients-13-03363-s001.zip › nutrients-1348781-supplementary Table S2.pdf]

Supplementary Table S2. Sociodemographic and lifestyle characteristics of Epirus Health Study participants by binary categories of Trail Making-Part B scores.

**Variables**	**Trail Making-Part B binary score**		**p value**
	**Normal performance (n= 1.123)**	**Abnormal performance (n=13)**	
Age	47.78 ± 10.71	37.43 ± 30.18	0.001 [a]
Female	668 (59.48)	5 (38.46)	0.158 [b]
Education			0.225 [b]
Primary and secondary school*	76 (6.77)	0 (0)	
High school**	289 (25.73)	6 (46.15)	
Higher education***	758 (67.50)	7 (53.85)	
MEDAS score	7.25 ± 1.75	6.54 ± 1.98	0.146 [a]
BMI	26.38 ± 4.68	24.03 ± 3.72	0.071 [a]
Smoking status			0.195 [b]
Non-smokers	500 (44.52)	5 (38.46)	
Former smokers	265 (23.60)	1 (7.69)	
Current smokers	358 (31.88)	7 (53.85)	
Alcohol consumption			0.563 [b]
Never	138 (12.29)	0 (0)	
Less than once/month	331 (29.47)	6 (46.15)	
1-3 times/month	187 (16.65)	1 (7.69)	
1-2 times/week	318 (28.32)	4 (30.77)	
Almost every day	149 (13.27)	2 (15.38)	
Physical activity (METs)	15.53 ± 20.60	17.96 ± 21.63	0.673 [a]

Abbreviations: BMI; Body mass index, METs; Metabolic Equivalents of Energy Expenditure
*Elementary school or junior high school, up to 9 years of education. **High school, up to 12 years of education. ***University degree/MSc/PhD/Postdoc, more than 13 years of education.
[a] Comparisons using t-test. [b] Comparison's using Fisher's exact test.
Mean ± standard deviation and frequency (percentage) are presented for continuous and categorical variables, respectively.
