# Supplementary material for: Adherence to Mediterranean Diet and Cognitive Abilities in the Greek Cohort of Epirus Health Study
Source: Nutrients. 2021 Sep 25;13(10):3363. doi: 10.3390/nu13103363 (PMC8541267; doi:10.3390/nu13103363)
Supplement: Supplementary file 1 [file nutrients-13-03363-s001.zip › nutrients-1348781-supplementary Table S3.pdf]

Supplementary Table S3. Sociodemographic and lifestyle characteristics of Epirus Health Study participants by binary categories of Verbal Fluency-semantic category scores.

**Variables**	**Verbal Fluency-semantic category score**		**p value**
	**Normal performance (n= 987)**	**Abnormal performance (n=60)**	
Age	47.82 ± 10.87	49.20 ± 13.39	0.347 [a]
Female	583 (59.07)	33 (55.00)	0.534 [b]
Education			0.090 [b]
Primary and secondary school*	72 (7.30)	9 (15.00)	
High school**	248 (25.15)	15 (25.00)	
Higher education***	666 (67.55)	36 (60.00)	
MEDAS score	7.26 ± 1.77	6.78 ± 1.55	0.043 [a]
BMI	26.40 ± 4.61	27.01 ± 5.45	0.339 [a]
Smoking status			0.531 [b]
Non-smokers	434 (43.97)	29 (48.33)	
Former smokers	244 (24.72)	11 (18.33)	
Current smokers	309 (31.31)	20 (33.33)	
Alcohol consumption			0.150 [c]
Never	119 (12.06)	10 (16.67)	
Less than once/month	279 (28.27)	23 (38.33)	
1-3 times/month	179 (18.14)	5 (8.33)	
1-2 times/week	280 (28.37)	16 (26.67)	
Almost every day	130 (13.17)	6 (10.00)	
Physical activity (METs)	16.06 ± 21.06	12.31 ± 19.16	0.178 [a]

Abbreviations: BMI; Body mass index, METs; Metabolic Equivalents of Energy Expenditure

*Elementary school or junior high school, up to 9 years of education. **High school, up to 12 years of education. ***University degree/MSc/PhD/Postdoc, more than 13 years of education.

[a] Comparisons using t-test. [b] Comparisons using x^2 test. [c] Comparison's using Fisher's exact test.

Mean ± standard deviation and frequency (percentage) are presented for continuous and categorical variables, respectively.
