# Supplementary material for: Adherence to Mediterranean Diet and Cognitive Abilities in the Greek Cohort of Epirus Health Study
Source: Nutrients. 2021 Sep 25;13(10):3363. doi: 10.3390/nu13103363 (PMC8541267; doi:10.3390/nu13103363)
Supplement: Supplementary file 1 [file nutrients-13-03363-s001.zip › nutrients-1348781-supplementary Table S4.pdf]

Supplementary Table S4. Sociodemographic and lifestyle characteristics of Epirus Health Study participants by binary categories of Verbal Fluency-phonemic category scores.

**Variables**	**Verbal Fluency-phonemic category score**		**p value**
	**Normal performance (n= 771)**	**Abnormal performance (n=263)**	
Age	47.47 ± 11.12	49.11 ± 10.57	0.037 [a]
Female	471 (61.09)	139 (52.85)	0.019 [b]
Education			0.042 [b]
Primary and secondary school*	69 (8.96)	11 (4.18)	
High school**	193 (25.06)	67 (25.48)	
Higher education***	508 (65.97)	185 (70.34)	
MEDAS score	7.19 ± 1.76	7.36 ± 1.77	0.163 [a]
BMI	26.40 ± 4.79	26.49 ± 4.32	0.769 [a]
Smoking status			0.202 [b]
Non-smokers	353 (45.78)	107 (40.68)	
Former smokers	178 (23.09)	74 (28.14)	
Current smokers	240 (31.13)	82 (31.18)	
Alcohol consumption			0.761 [b]
Never	97 (12.58)	29 (11.03)	
Less than once/month	226 (29.31)	72 (27.38)	
1-3 times/month	132 (17.12)	51 (19.39)	
1-2 times/week	220 (28.53)	73 (27.76)	
Almost every day	96 (12.45)	38 (14.45)	
Physical activity (METs)	16.47 ± 20.92	14.20 ± 21.36	0.130 [a]

Abbreviations: BMI; Body mass index, METs; Metabolic Equivalents of Energy Expenditure
*Elementary school or junior high school, up to 9 years of education. **High school, up to 12 years of education. ***University degree/MSc/PhD/Postdoc, more than 13 years of education.
[a] Comparisons using t-test. [b] Comparisons using x^2 test.
Mean ± standard deviation and frequency (percentage) are presented for continuous and categorical variables, respectively.
