# Supplementary material for: Adherence to Mediterranean Diet and Cognitive Abilities in the Greek Cohort of Epirus Health Study
Source: Nutrients. 2021 Sep 25;13(10):3363. doi: 10.3390/nu13103363 (PMC8541267; doi:10.3390/nu13103363)
Supplement: Supplementary file 1 [file nutrients-13-03363-s001.zip › nutrients-1348781-supplementary Table S5.pdf]

Supplementary Table S5. Sociodemographic and lifestyle characteristics of Epirus Health Study participants by binary categories of Logical Memory-immediate recall scores.

**Variables**	**Logical Memory-immediate recall score**		**p value**
	**Normal performance (n= 1.147)**	**Abnormal performance (n=49)**	
Age	47.86 ± 11.01	47.14 ± 11.02	0.658 [a]
Female	689 (60.07)	22 (44.90)	0.034 [b]
Education			0.156 [c]
Primary and secondary school*	81 (7.07)	5 (10.20)	
High school**	289 (25.22)	17 (34.69)	
Higher education***	776 (67.71)	27 (55.10)	
MEDAS score	7.26 ± 1.73	6.98 ± 2.06	0.270 [a]
BMI	26.37 ± 4.62	27.36 ± 6.09	0.152 [a]
Smoking status			0.349 [b]
Non-smokers	511 (44.55)	20 (40.82)	
Former smokers	276 (24.06)	9 (18.37)	
Current smokers	360 (31.39)	20 (40.82)	
Alcohol consumption			0.811 [b]
Never	139 (12.12)	8 (16.33)	
Less than once/month	340 (29.64)	12 (24.49)	
1-3 times/month	197 (17.18)	8 (16.33)	
1-2 times/week	323 (28.16)	13 (26.53)	
Almost every day	148 (12.90)	8 (16.33)	
Physical activity (METs)	15.51 ± 20.13	16.80 ± 9.86	0.663 [a]

Abbreviations: BMI; Body mass index, METs; Metabolic Equivalents of Energy Expenditure

*Elementary school or junior high school, up to 9 years of education. **High school, up to 12 years of education. ***University degree/MSc/PhD/Postdoc, more than 13 years of education.

[a] Comparisons using t-test. [b] Comparisons using x^2 test. [c] Comparison's using Fisher's exact test.

Mean ± standard deviation and frequency (percentage) are presented for continuous and categorical variables, respectively.
