# Supplementary material for: Adherence to Mediterranean Diet and Cognitive Abilities in the Greek Cohort of Epirus Health Study
Source: Nutrients. 2021 Sep 25;13(10):3363. doi: 10.3390/nu13103363 (PMC8541267; doi:10.3390/nu13103363)
Supplement: Supplementary file 1 [file nutrients-13-03363-s001.zip › nutrients-1348781-supplementary Table S6.pdf]

Supplementary Table S6. Sociodemographic and lifestyle characteristics of Epirus Health Study participants by binary categories of Logical Memory-delayed recall scores.

**Variables**	**Logical Memory-delayed recall score**		**p value**
	**Normal performance (n= 1.168)**	**Abnormal performance (n=28)**	
Age	47.71 ± 10.95	52.71 ± 12.56	0.017 [a]
Female	697 (59.67)	14 (50.00)	0.303 [b]
Education			4.029^{-04} [b]
Primary and secondary school*	79 (6.77)	7 (25.00)	
High school**	297 (25.45)	9 (32.14)	
Higher education***	791 (67.78)	12 (42.86)	
MEDAS score	7.26 ± 1.75	7.00 ± 1.59	0.445 [a]
BMI	26.38 ± 4.66	27.84 ± 5.76	0.109 [a]
Smoking status			0.220 [c]
Non-smokers	517 (44.26)	14 (50.00)	
Former smokers	276 (23.63)	9 (32.14)	
Current smokers	375 (32.11)	5 (17.86)	
Alcohol consumption			0.122 [c]
Never	141 (12.07)	6 (21.43)	
Less than once/month	345 (29.54)	7 (25.00)	
1-3 times/month	203 (17.38)	2 (7.14)	
1-2 times/week	330 (28.25)	6 (21.43)	
Almost every day	149 (12.76)	7 (25.00)	
Physical activity (METs)	15.53 ± 20.26	16.71 ± 22.31	0.761 [a]

Abbreviations: BMI; Body mass index, METs; Metabolic Equivalents of Energy Expenditure
*Elementary school or junior high school, up to 9 years of education. **High school, up to 12 years of education. ***University degree/MSc/PhD/Postdoc, more than 13 years of education.
[a] Comparisons using t-test. [b] Comparisons using x^2 test. [c] Comparison's using Fisher's exact test.
Mean ± standard deviation and frequency (percentage) are presented for continuous and categorical variables, respectively.
