# Supplementary material for: Adherence to Mediterranean Diet and Cognitive Abilities in the Greek Cohort of Epirus Health Study
Source: Nutrients. 2021 Sep 25;13(10):3363. doi: 10.3390/nu13103363 (PMC8541267; doi:10.3390/nu13103363)
Supplement: Supplementary file 1 [file nutrients-13-03363-s001.zip › nutrients-1348781-supplementary Table S7.pdf]

Supplementary Table S7. Results of ordinal logistic regression for the associations between adherence to Mediterranean diet (continuous score) and cognitive function (ordinal score) among Epirus Health Study participants.

**Cognitive scores †**	**Adherence to Mediterranean diet ‡**			
	**Model 1[a]**		**Model 2[b]**	
	**OR**	**95% CI**	**OR**	**95% CI**
Trail Making Test (Part A)	1.036	0.957, 1.121	1.027	0.946, 1.114
Trail Making Test (Part B)	1.025	0.939, 1.118	1.039	0.950, 1.138
Verbal Fluency (semantic)	0.951	0.876, 1.032	0.966	0.885, 1.053
Verbal Fluency (phonemic)	1.024	0.957, 1.095	1.028	0.958, 1.103
Logical Memory (immediate recall)	1.014	0.941, 1.092	1.027	0.950, 1.110
Logical Memory (delayed recall)	1.011	.939, 1.088	1.033	0.955, 1.116

[a] Adjusted for age, gender, education level. [b] Adjusted for age, gender, education level, BMI, smoking status, alcohol consumption and physical activity.

† Cognitive scores analyzed as ordinal score (normal performance defined as scores at the mean value and above, borderline performance defined as scores up to 1.5 standard deviation below the mean value, and abnormal performance defined as scores lower than 1.5 standard deviation below the mean)

‡Adherence to Mediterranean diet was assessed using the Mediterranean Diet Adherence Screener (MEDAS). MEDAS score analyzed as continuous score (0-14).
