# Supplementary material for: Adherence to Mediterranean Diet and Cognitive Abilities in the Greek Cohort of Epirus Health Study
Source: Nutrients. 2021 Sep 25;13(10):3363. doi: 10.3390/nu13103363 (PMC8541267; doi:10.3390/nu13103363)
Supplement: Supplementary file 1 [file nutrients-13-03363-s001.zip › nutrients-1348781-supplementary Table S8.pdf]

Supplementary Table S8. Results of logistic regression for the associations between adherence to Mediterranean diet (continuous score) and cognitive function (binary score) among Epirus Health Study participants.

**Cognitive scores †**	**Adherence to Mediterranean diet ‡**			
	**Model 1[a]**		**Model 2[b]**	
	**OR**	**95% CI**	**OR**	**95% CI**
Trail Making Test (Part A)	1.013	0.815, 1.258	1.012	0.806, 1.272
Trail Making Test (Part B)	0.927	0.667, 1.287	0.900	0.641, 1.263
Verbal Fluency (semantic)	0.845	0.724, 0.985	0.849	0.721, 0.999
Verbal Fluency (phonemic)	1.036	0.952, 1.127	1.057	0.968, 1.155
Logical Memory (immediate recall)	0.945	0.798, 1.118	0.976	0.820, 1.162
Logical Memory (delayed recall)	0.890	0.715, 1.107	0.893	0.710, 1.122

[a] Adjusted for age, gender, education level. [b] Adjusted for age, gender, education level, BMI, smoking status, alcohol consumption and physical activity.

† Cognitive scores analyzed as binary score (normal performance defined as performance better than 1.5 standard deviation below the mean value, and abnormal performance defined as scores lower than 1.5 standard deviation below the mean)

‡Adherence to Mediterranean diet was assessed using the Mediterranean Diet Adherence Screener (MEDAS). MEDAS score analyzed as continuous score (0-14).
