# Supplementary material for: Adherence to Mediterranean Diet and Cognitive Abilities in the Greek Cohort of Epirus Health Study
Source: Nutrients. 2021 Sep 25;13(10):3363. doi: 10.3390/nu13103363 (PMC8541267; doi:10.3390/nu13103363)
Supplement: Supplementary file 1 [file nutrients-13-03363-s001.zip › nutrients-1348781-supplementary Table S9.pdf]

Supplementary Table S9. Results of multivariable linear regression for the associations between adherence to Mediterranean diet (binary score) and cognitive function (continuous score) among Epirus Health Study participants.

Cognitive scores	Adherence to Mediterranean diet †			
	Model 1[a]		Model 2[b]	
	β	95% CI	β	95% CI
Trail Making Test (Part A)	-0.016	-1.365, 1.334	-0.349	-1.731, 1.033
Trail Making Test (Part B)	-0.390	-2.368, 1.588	-0.690	-2.721, 1.342
Trail Making Test (Part B-A)	-0.646	-2.359, 1.068	-0.621	-2.382, 1.140
Verbal Fluency (semantic)	0.551	-0.161, 1.262	0.534	-0.205, 1.274
Verbal Fluency (phonemic)	-0.125	-0.577, 0.328	-0.239	-0.707, 0.230
Logical Memory (immediate recall)	-0.102	-0.676, 0.472	-0.228	-0.827, 0.370
Logical Memory (delayed recall)	0.047	-0.253, 0.347	-0.066	-0.376, 0.244
Composite z-score	0.032	-0.046, 0.110	0.017	-0.063, 0.098

[a] Adjusted for age, gender, education level. [b] Adjusted for age, gender, education level, BMI, smoking status, alcohol consumption and physical activity.

† Adherence to Mediterranean diet was assessed using the Mediterranean Diet Adherence Screener (MEDAS). MEDAS score analyzed as binary categories of low and high adherence, ranged between 0-7 and 8-14, respectively.
